# Supplementary material for: Optical and Electrochemical Properties of a Photosensitive Pyromellitic Diimide Derivative of Cymantrene
Source: Molecules. 2023 Oct 15;28(20):7098. doi: 10.3390/molecules28207098 (PMC10608918; doi:10.3390/molecules28207098)
Supplement: Supplementary file 1 [file molecules-28-07098-s001.zip › molecules-2664791-supplementary.pdf]

Supporting Information

Table of Contents

IR spectra. 3
NMR spectra. 5
MS spectra. 7
UV-vis spectra. 10
Kinetic study. 11
Cyclic voltammograms. 13
DFT study. 14

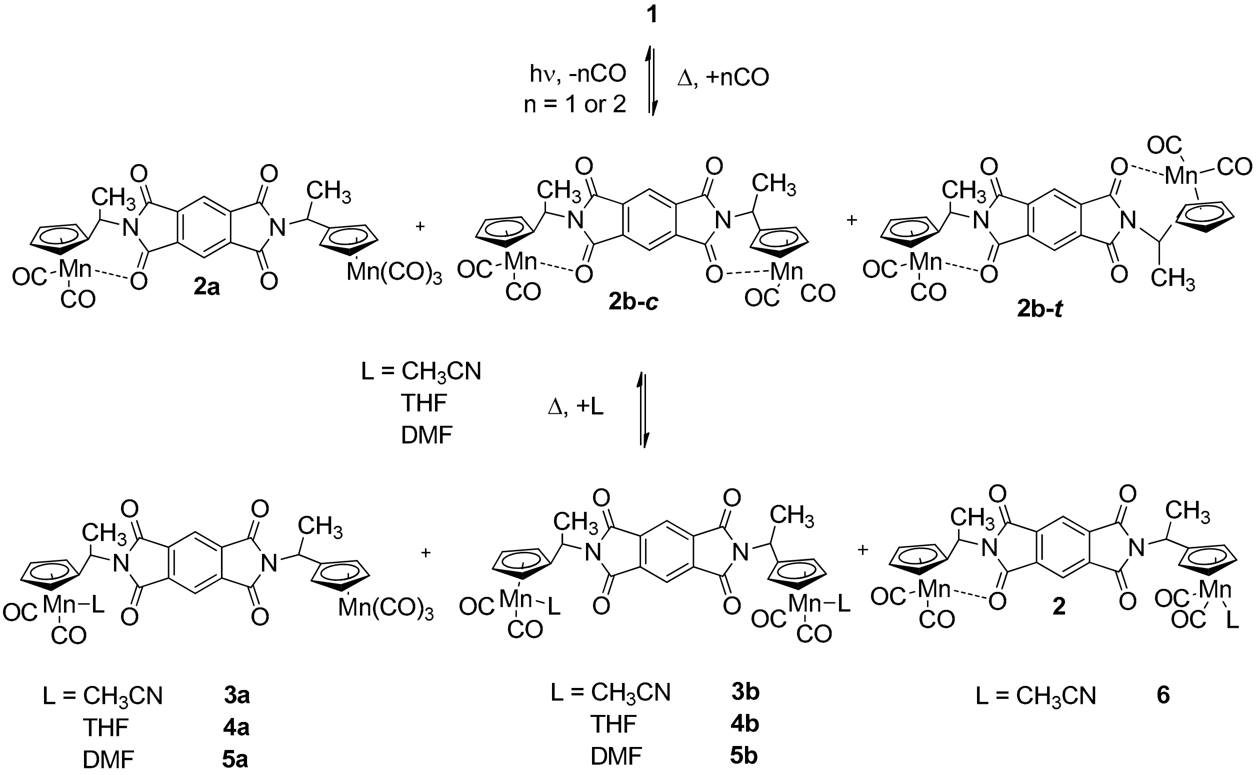

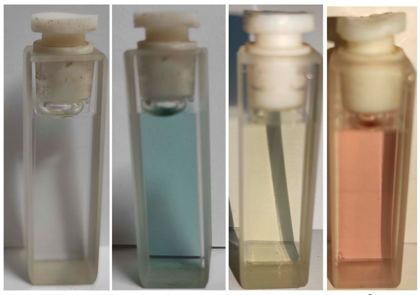

Scheme S1. Stage 1 – photolysis of compound **1** in benzene. Stage 2 – thermal reaction of compound **1** in the presence of donor ligands. Color of benzene solutions for compounds **1-2**, color of acetonitrile solutions for compound **3**, color of THF solutions for compound **4**.

IR spectra.

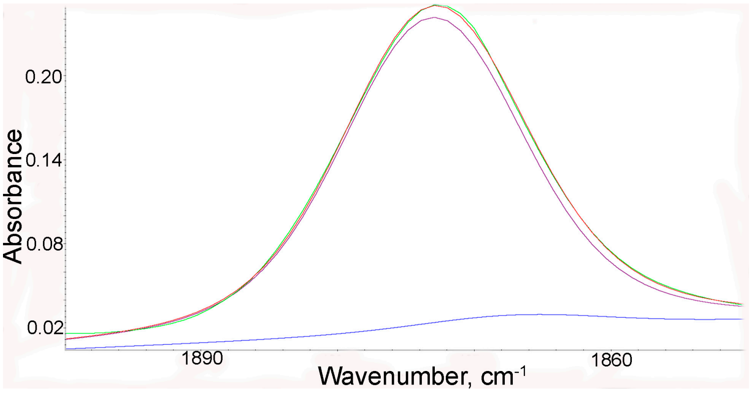

Figure S1. Curve fitting of the ν(CO) band of dicarbonyl complexes in the IR spectrum of benzene solution after irradiation of **1** with CH_3CN (4 eq.) for 4 min

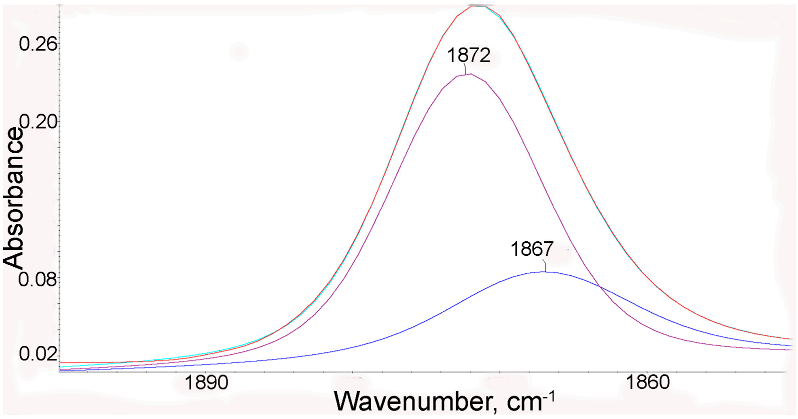

Figure S2. Curve fitting of the ν(CO) band of dicarbonyl complexes in the IR spectrum of benzene solution after irradiation of **1** and the subsequent thermal reaction for 90 min in closed IR cell at room temperature.

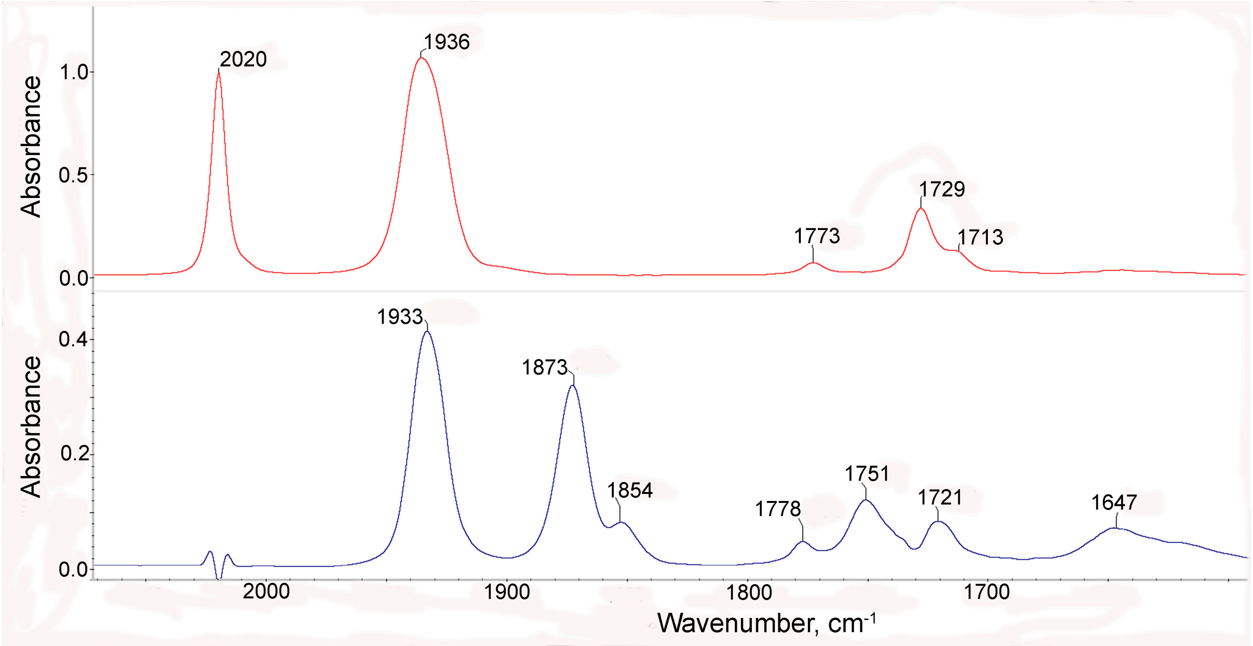

Figure S3. IR spectra in the ν(CO) range of a THF solution of **1** (red line) and the photolysis products (blue line) The latter was obtained by the subtraction of the spectrum of **1** from the spectrum of the reaction mixture after irradiation of **1** and represents the spectrum of the photolysis products.

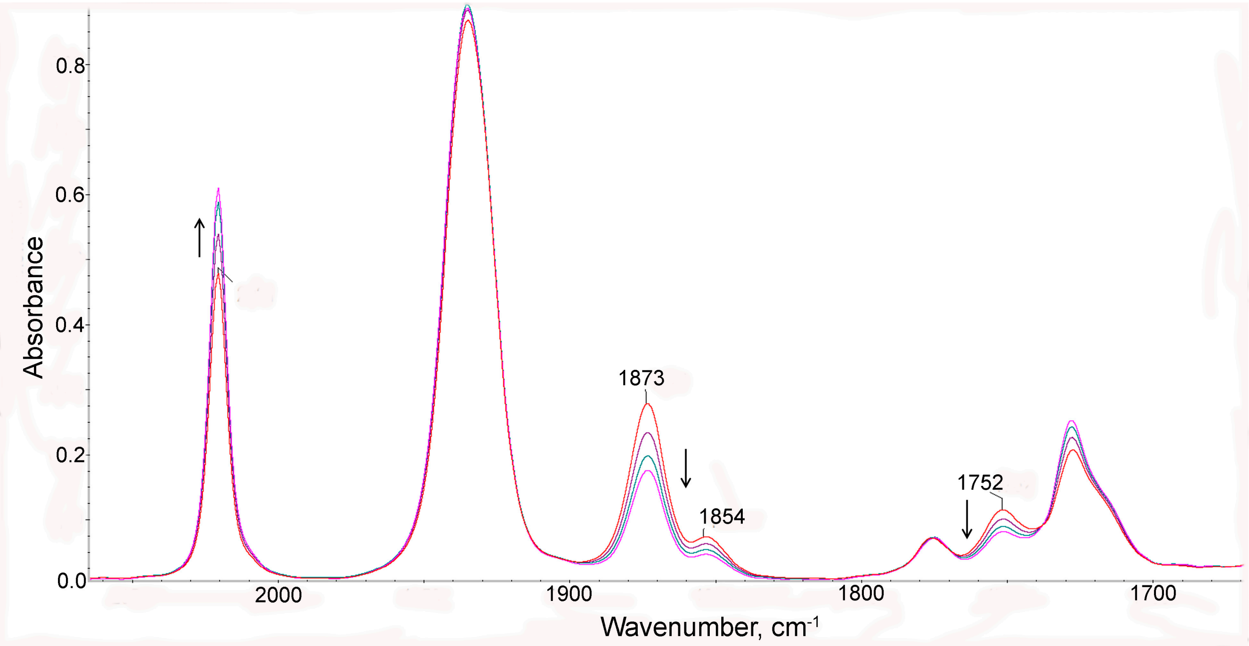

Figure S4. IR monitoring of the transformation **2** and **4** to **1** in the region of ν(CO) stretches after irradiation of **1** for 4 min at room temperature. The spectra were recorded every 5 min in a sealed IR cell.

NMR spectra.

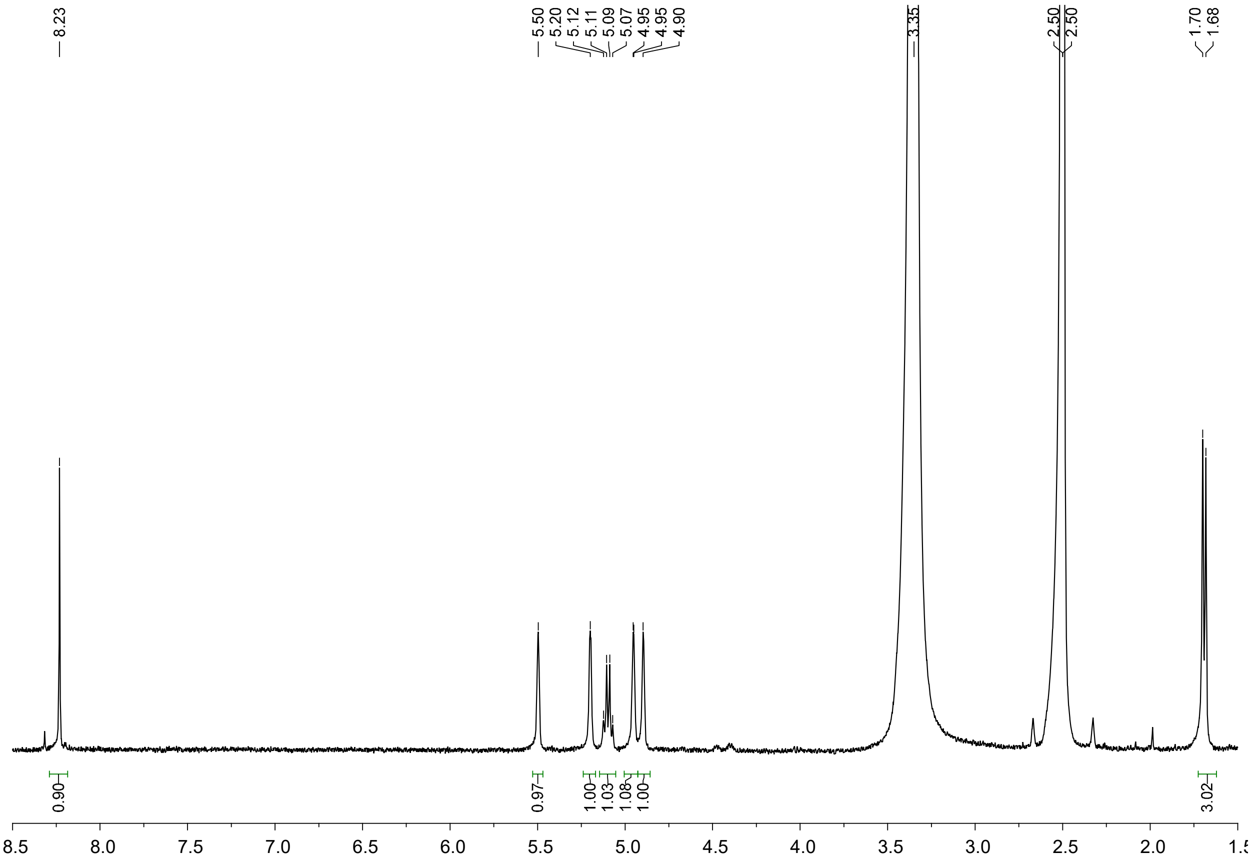

Figure S5. ^{1}H NMR spectrum of compound **1** in DMSO-d_6.

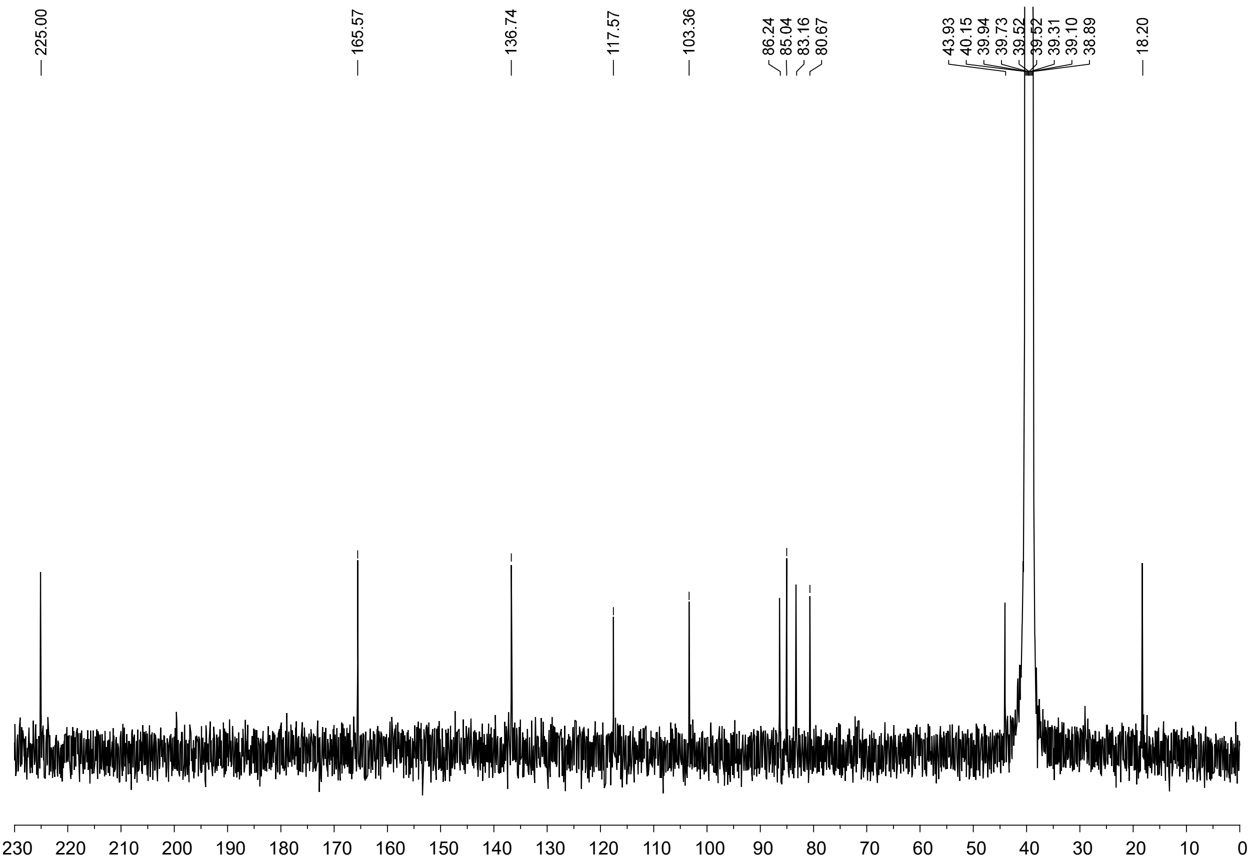

Figure S6. ^{13}C NMR spectrum of compound **1** in DMSO-d_6.

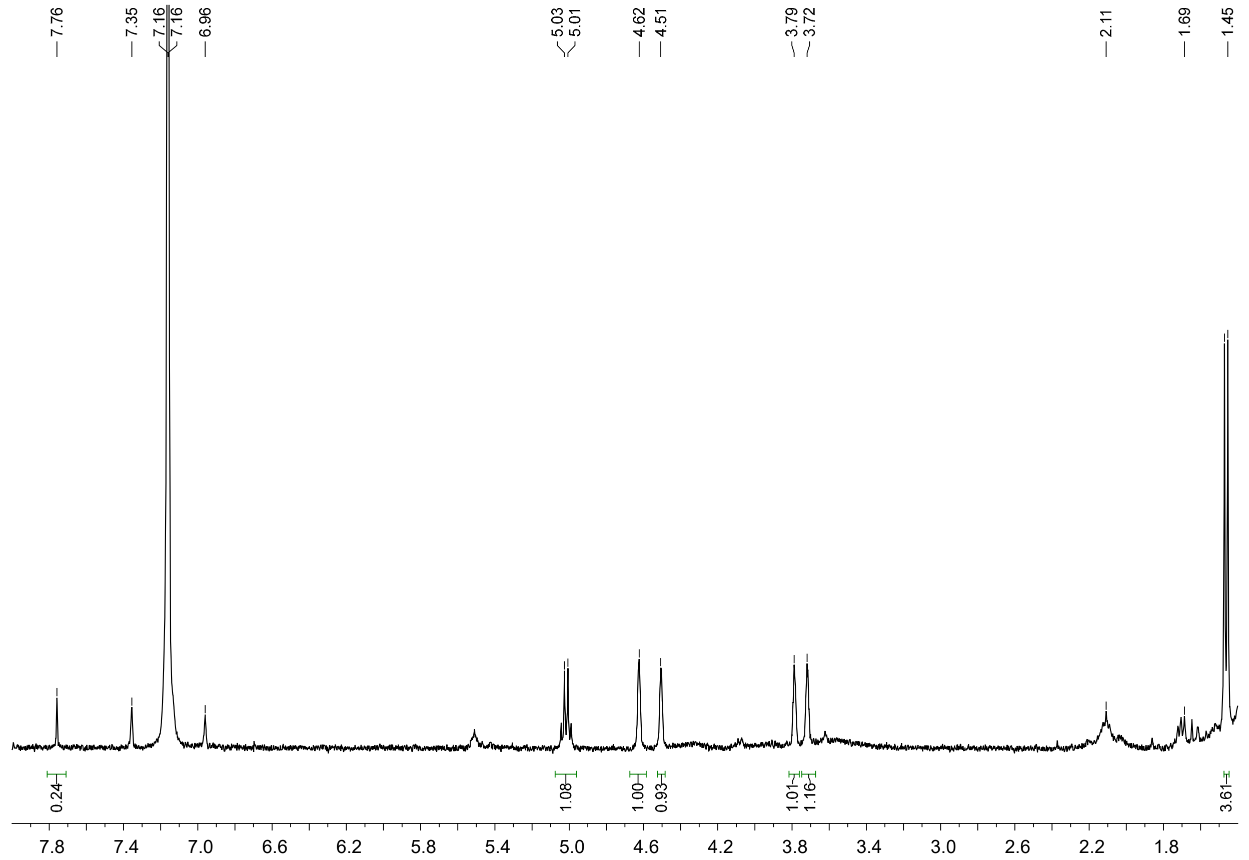

Figure S7. ^{1}H NMR spectrum of compound **1** in benzene-d_6 (256 scans).

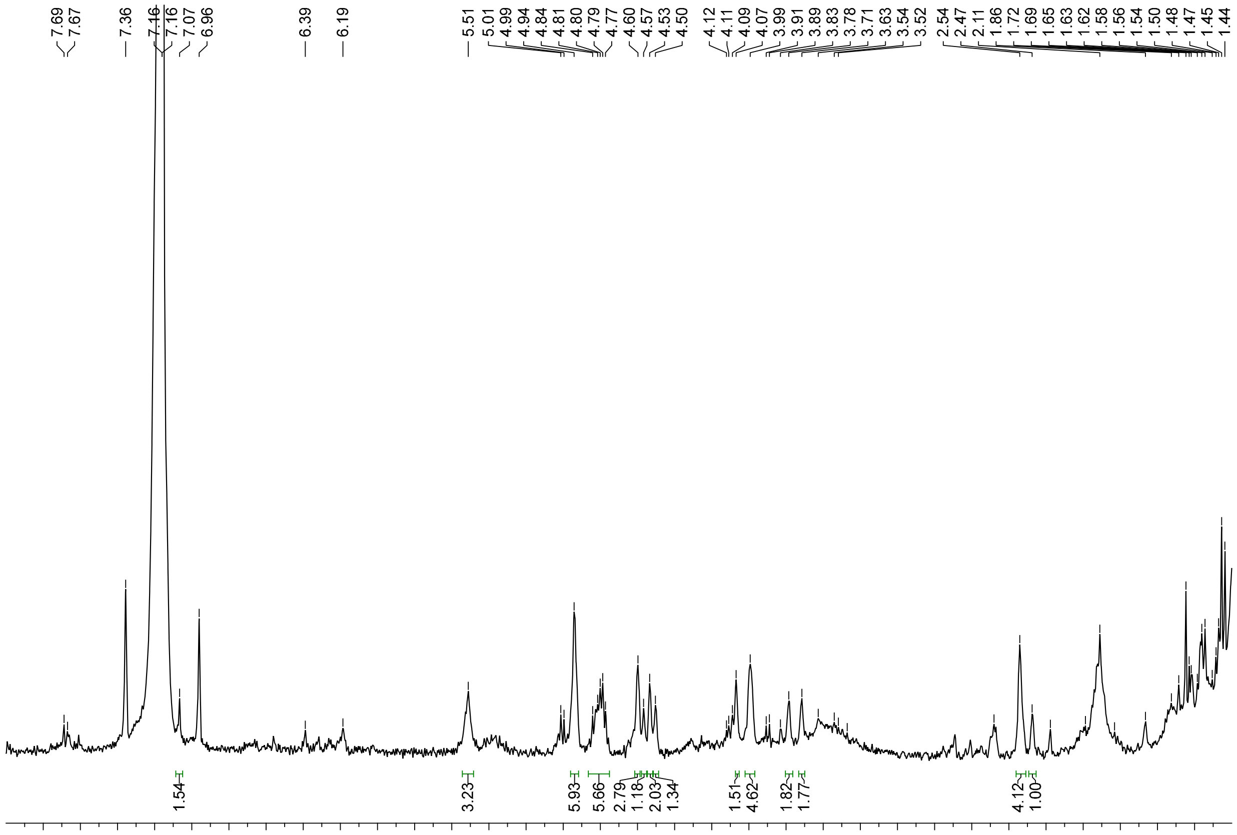

Figure S8. ^{1}H NMR spectrum of compound **1** in benzene-d6 after irradiation for 30 sec (256 scans).

MS spectra.

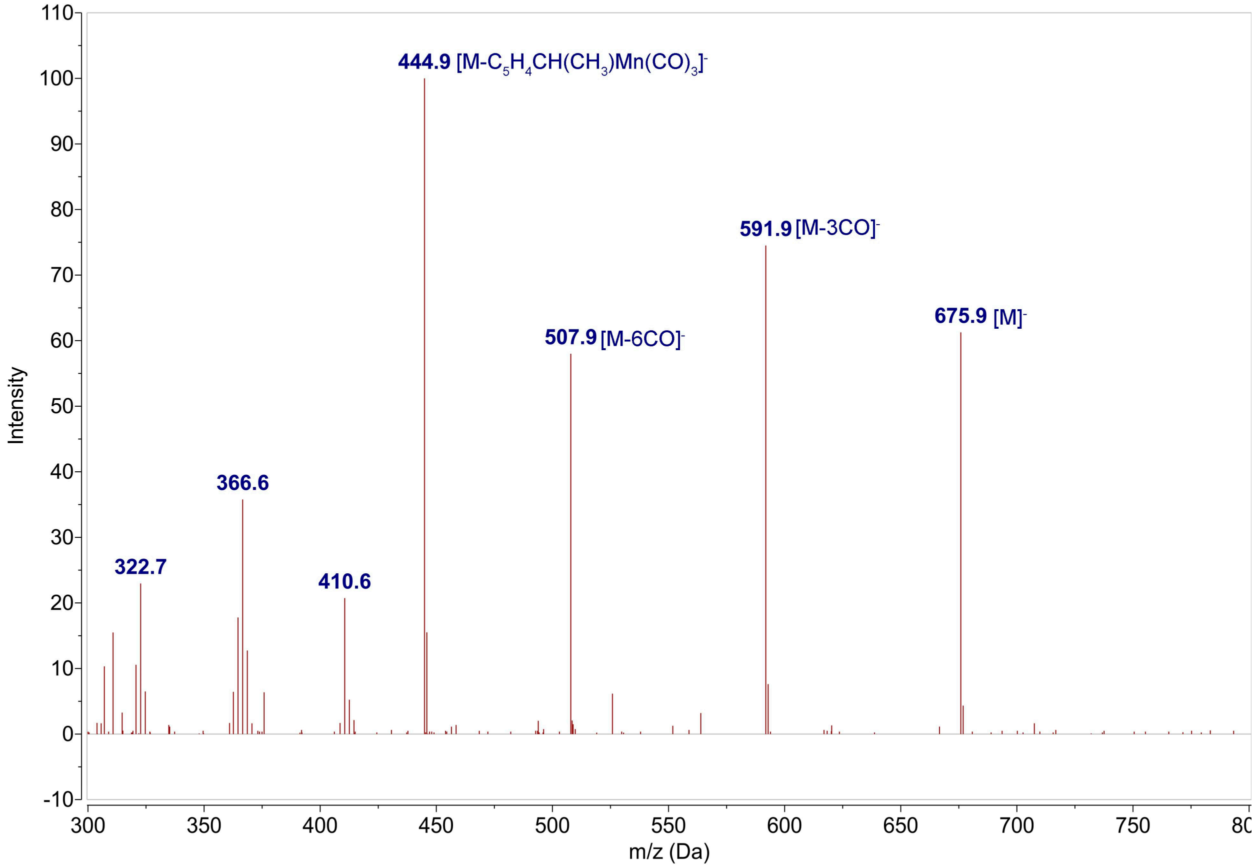

Figure S9. MALDI mass-spectrum of compound **1** (monitoring of photolysis of **1** in benzene).

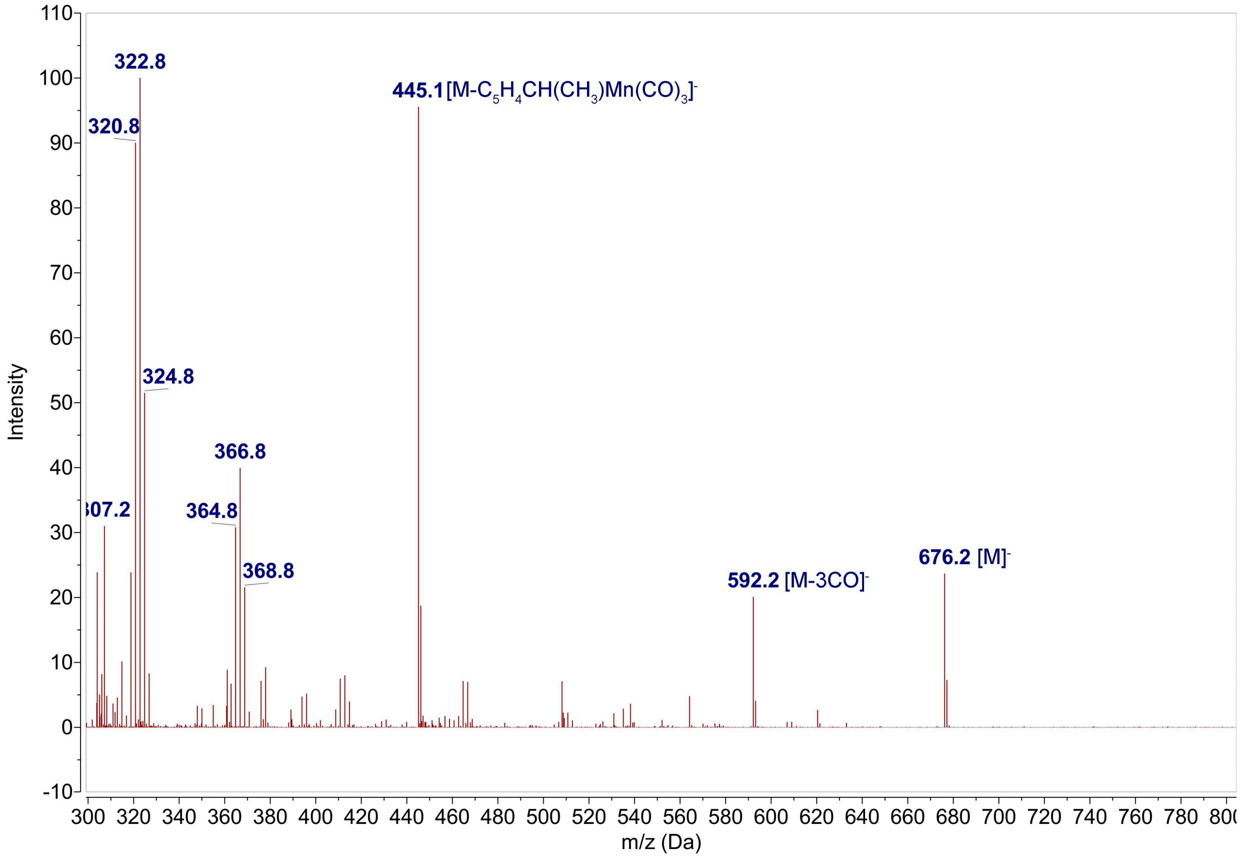

Figure S10. MALDI mass-spectrum of compound **1** after irradiation for 45 s in benzene.

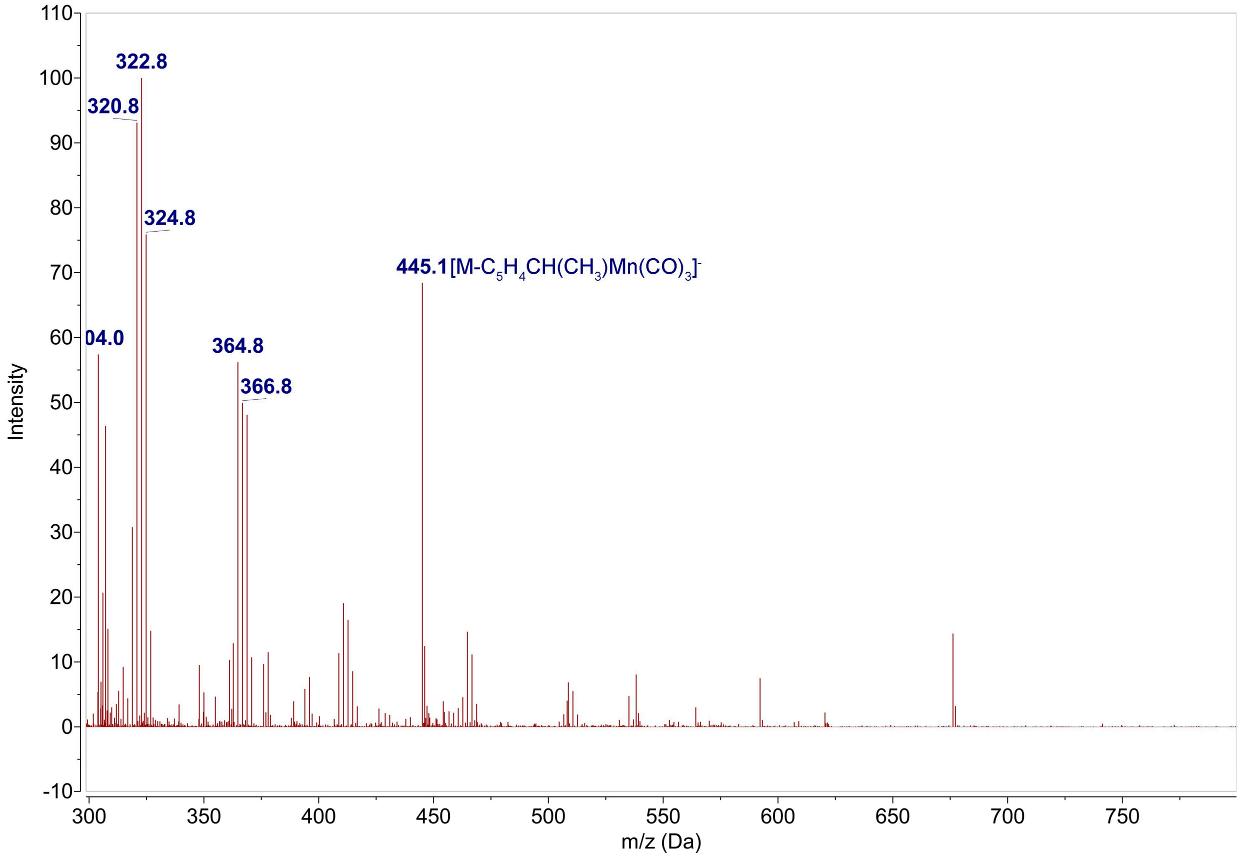

Figure S11. MALDI mass-spectrum of compound **1** after irradiation for 135 s in benzene.

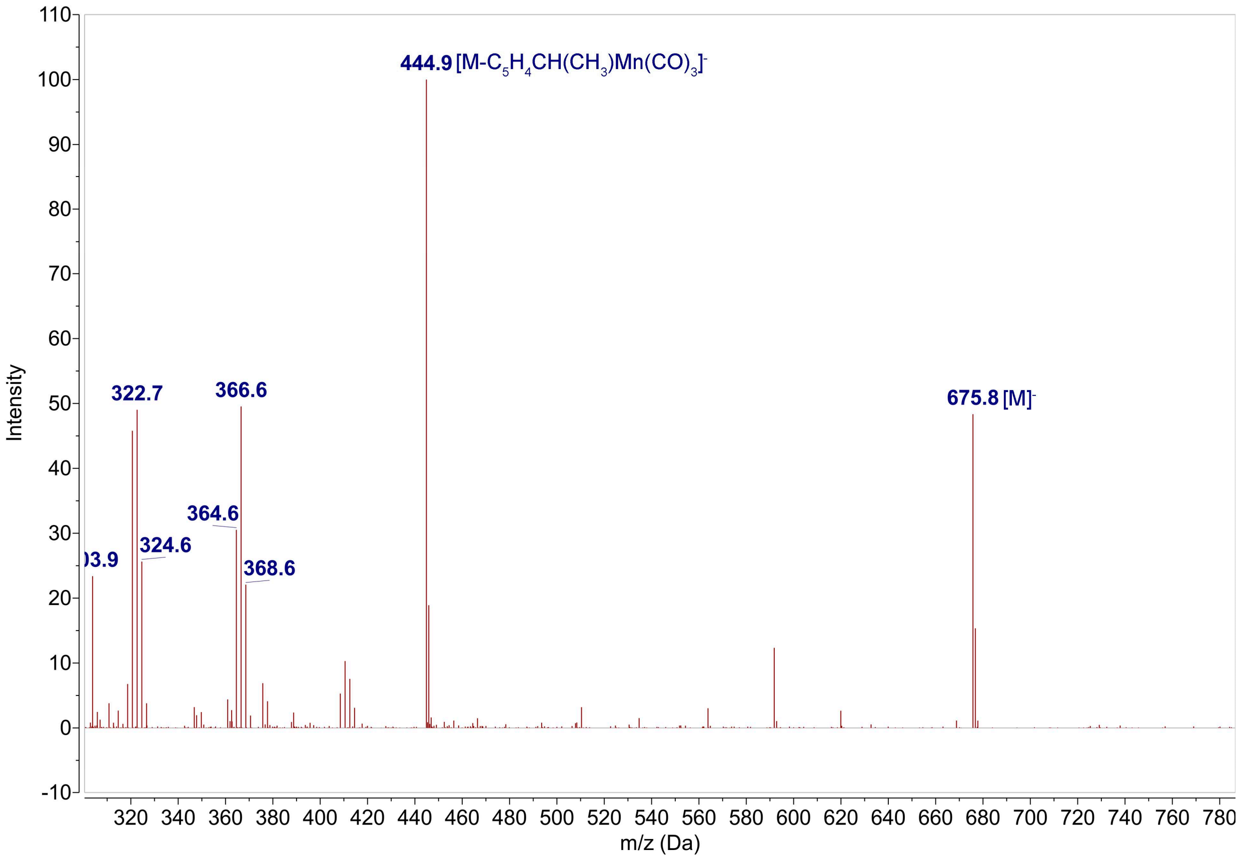

Figure S12. MALDII mass-spectrum of compound **1** (monitoring of photolysis of **1** in benzene : acetonitrile).

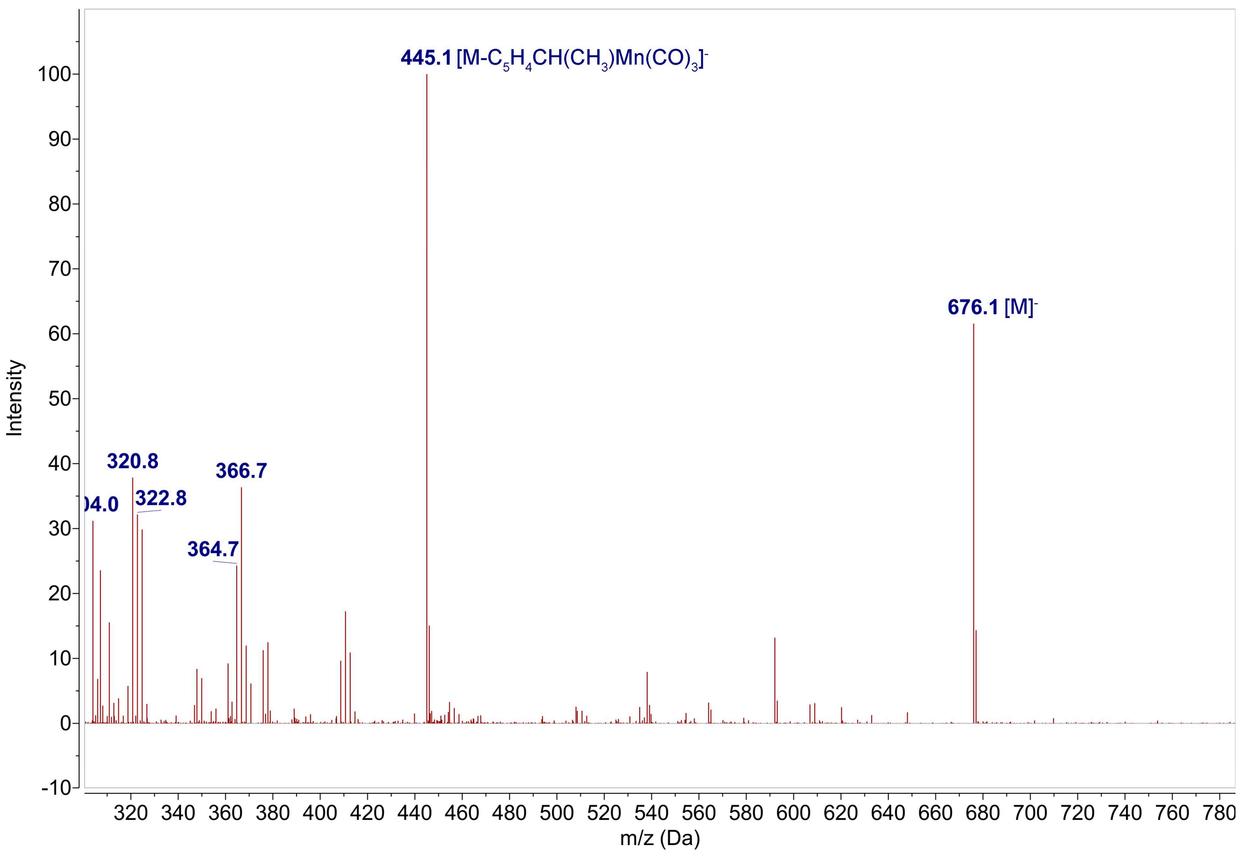

Figure S13. ESI mass-spectrum of compound **1** after irradiation for 45 s in benzene : acetonitrile.

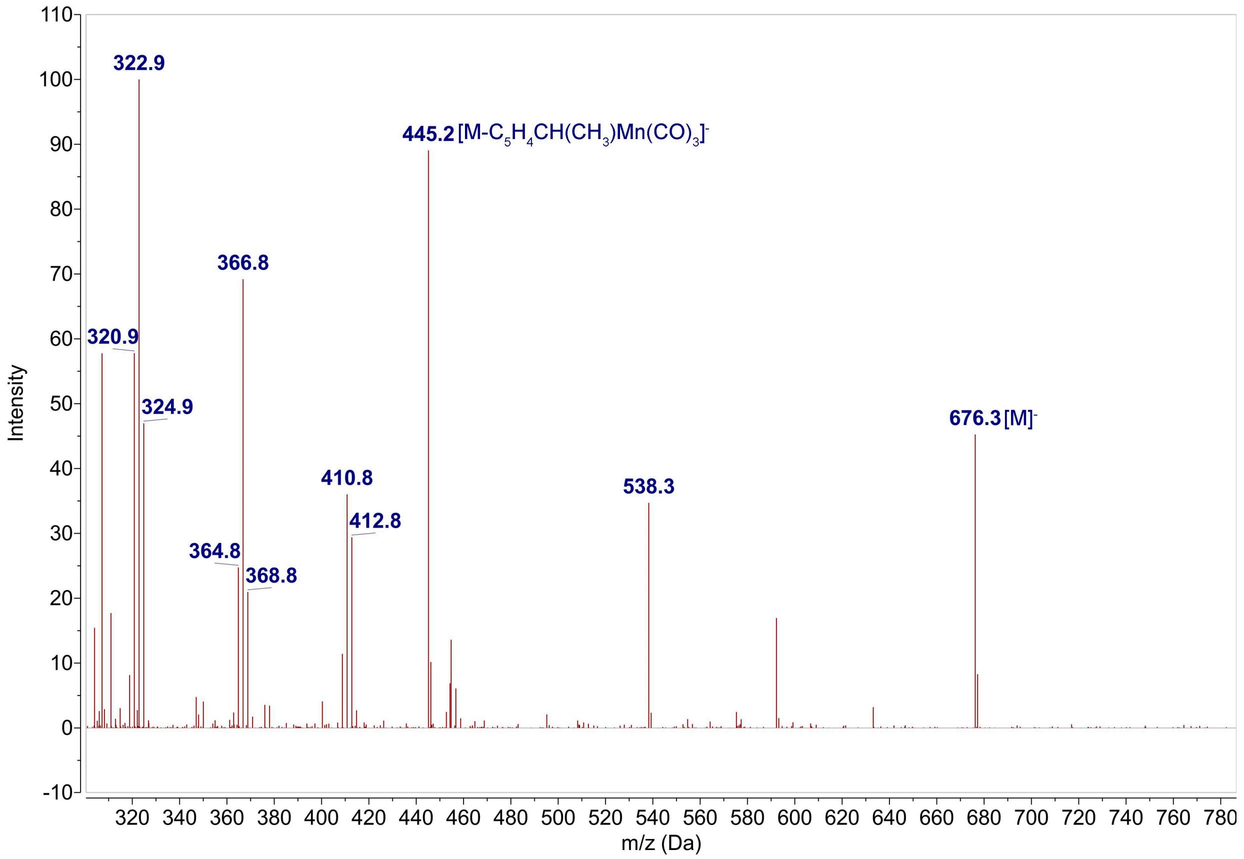

Figure S14. MALDI mass-spectrum of compound **1** after irradiation for 135 s in benzene : acetonitrile.

UV-vis spectra.

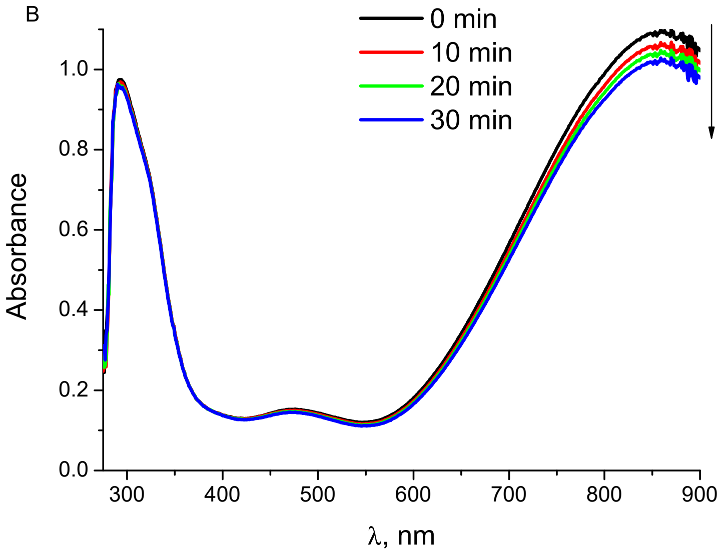

Figure S15. UV-vis monitoring of the thermal reversible reaction after irradiation of **1** in benzene during 30 min.

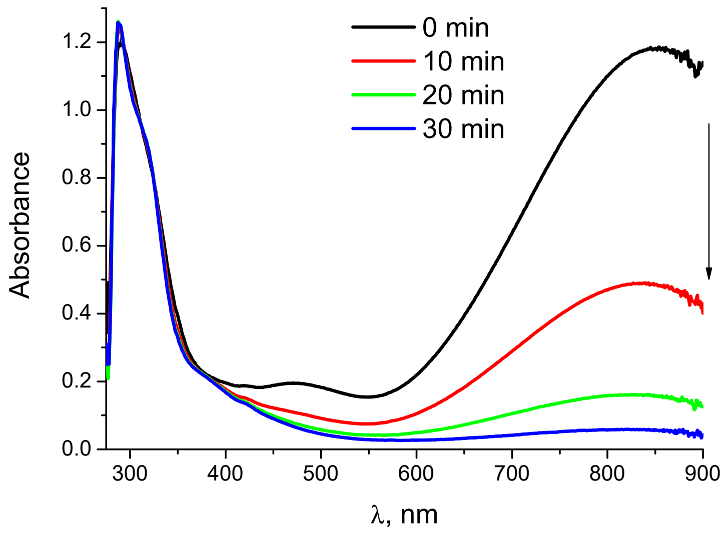

Figure S16. UV-vis monitoring of the thermal reaction after irradiation of **1** in acetonitrile during 30 min.

Kinetic study.

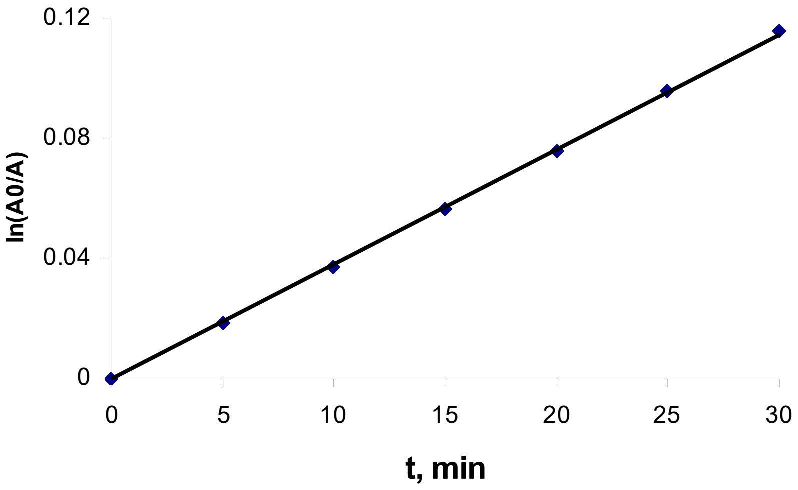

Figure S17. The kinetic curve for thermal transformation of chelates **2** to **1** (C 1 мM) in benzene at 24 ºC (R = 0.9997) by IR monitoring.

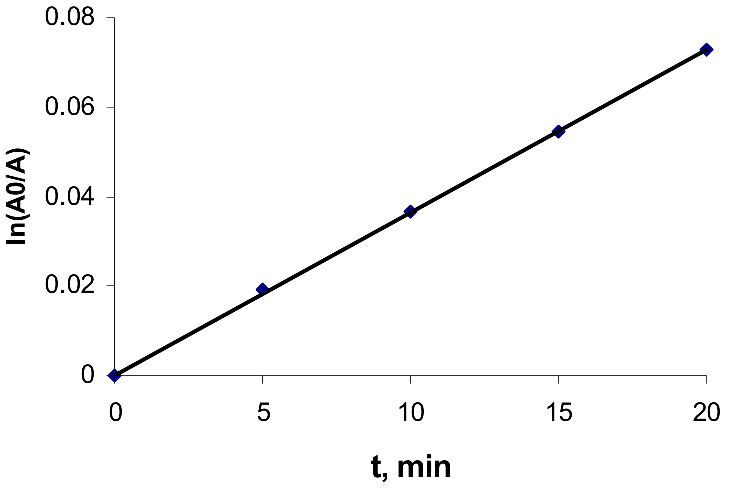

Figure S18. The kinetic curve for thermal transformation of chelates **2** to **1** (C 1 мM) in benzene at 24 ºC (R = 0.9991) at 859 nm by UV-vis monitoring.

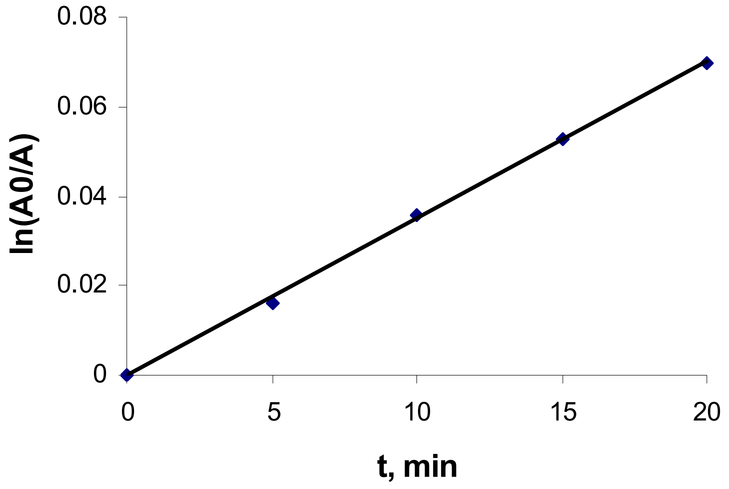

Figure S19. The kinetic curve for thermal transformation of chelates **2** to **1** (C 1 мM) in benzene at 24 ºC (R = 0.9995) at 475 nm by UV-vis monitoring.

Cyclic voltammograms.

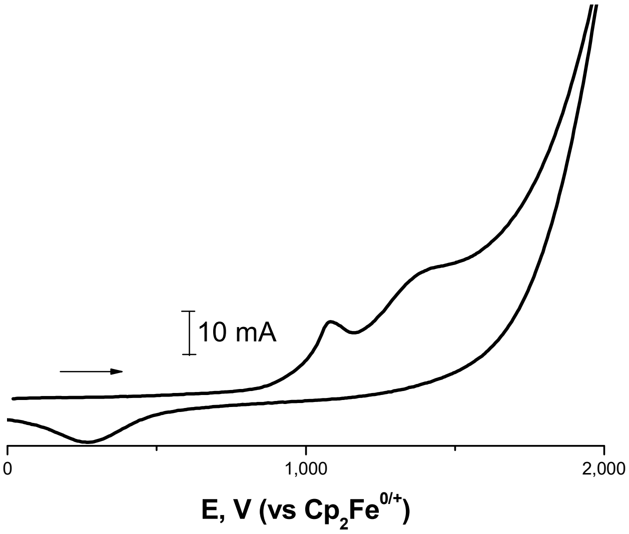

Figure S20. Cyclic voltammogram of compound **7** in 0.1 M tetraethylammonium tetrafluoroborate solution (relative to $Fc^{0/+}$). Scanning speed 100 mV/s.

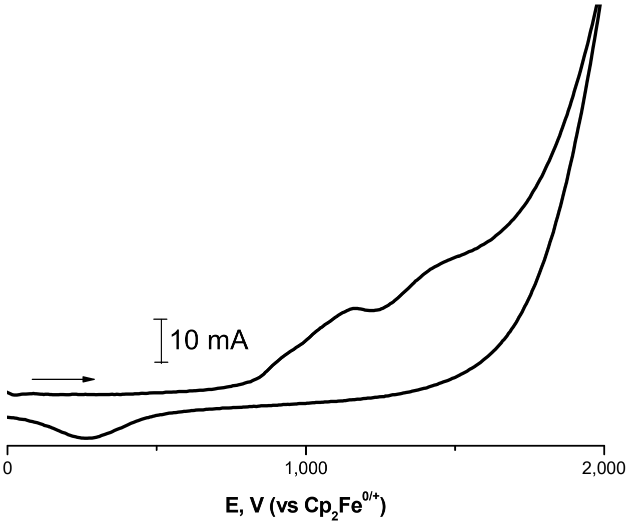

Figure S21. Cyclic voltammogram of compound **7** after irradiation for 2 min in 0.1 M tetraethylammonium tetrafluoroborate solution (relative to $Fc^{0/+}$). Scanning speed 100 mV/s.

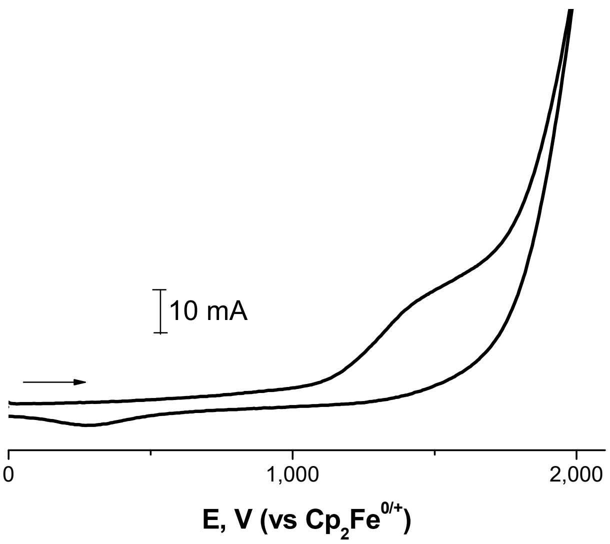

Figure S22. Cyclic voltammogram of compound **1** in 0.1 M tetraethylammonium tetrafluoroborate solution (relative to $Fc^{0/+}$). Scanning speed 100 mV/s.

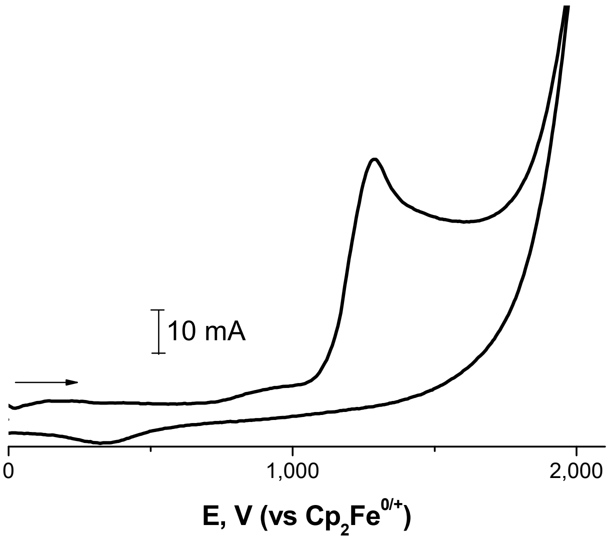

Figure S23. Cyclic voltammogram of compound **1** after irradiation for 2 min in 0.1 M tetraethylammonium tetrafluoroborate solution (relative to $Fc^{0/+}$). Scanning speed 100 mV/s.

DFT study.

Table S1. Selected TD-DFT results at B3lYB/Def2-TZVP level

Number of transition	L, nm	Oscillator strength	Transition orbitals
**1**			
10	352	0.03899	169→174

			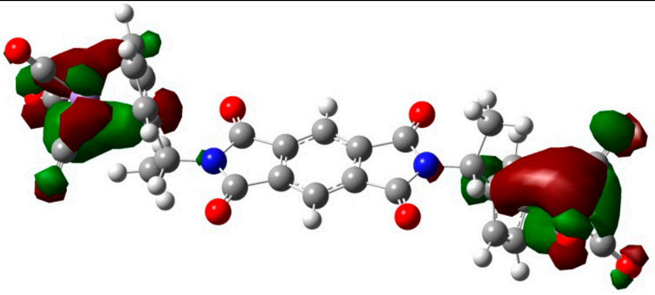 → 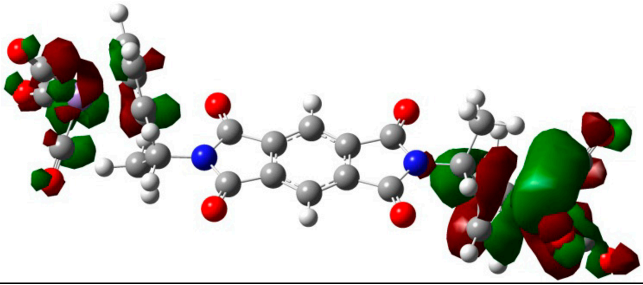
**2a**			
1	832	0.02049	162→164 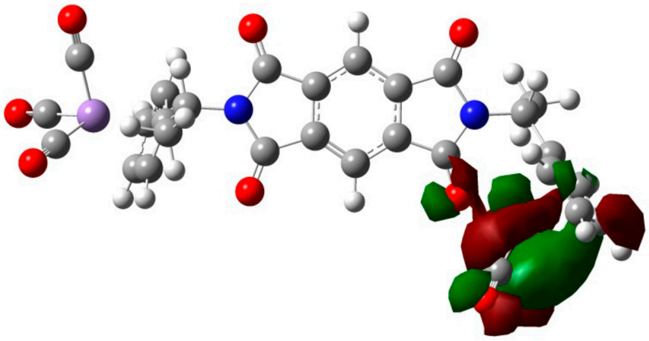 → 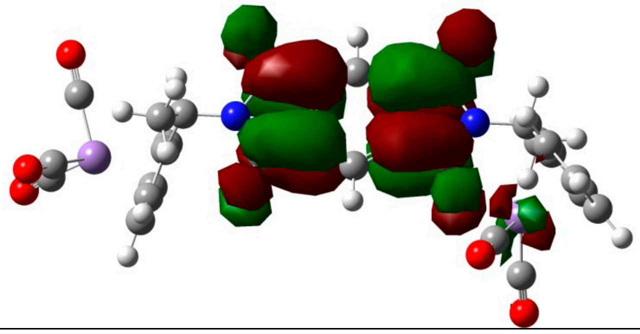
2	750	0.20162	163→164 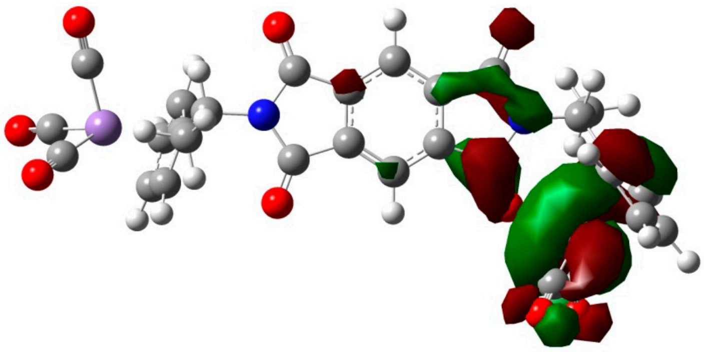 →

			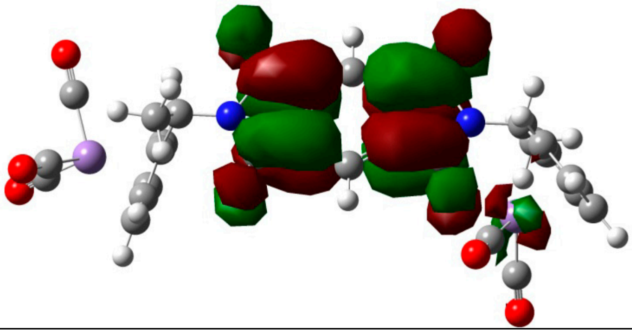
7	484	0.02409	163→165<br>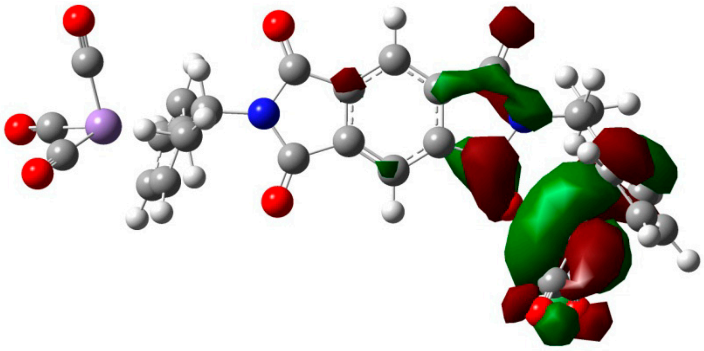<br>→<br>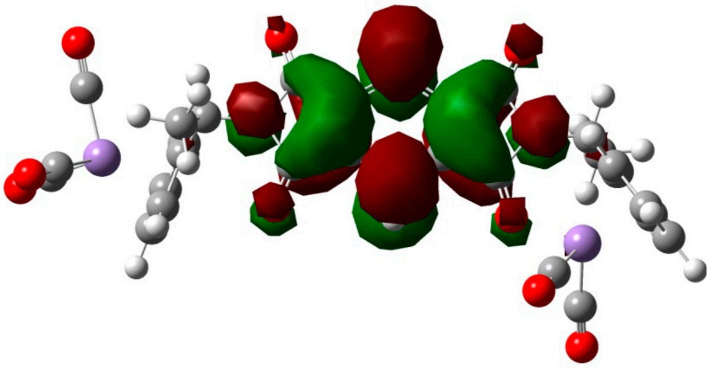
13	397	0.01203	157-164<br>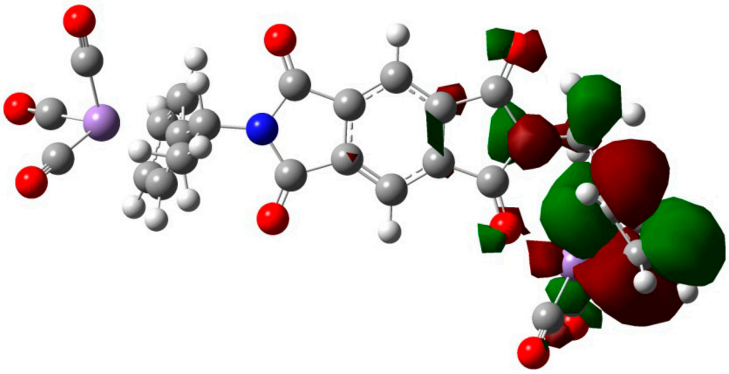<br>→<br>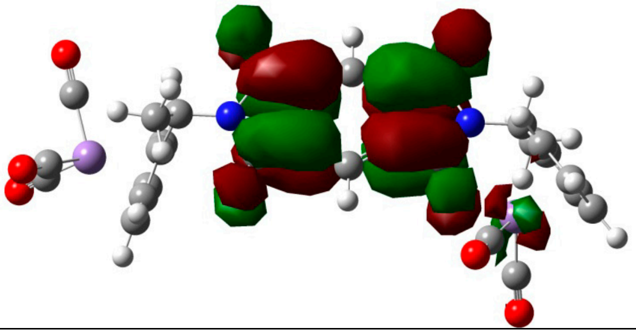
**2b-*t***			

1	873	0.16781	156 (HOMO) →157 (LUMO) 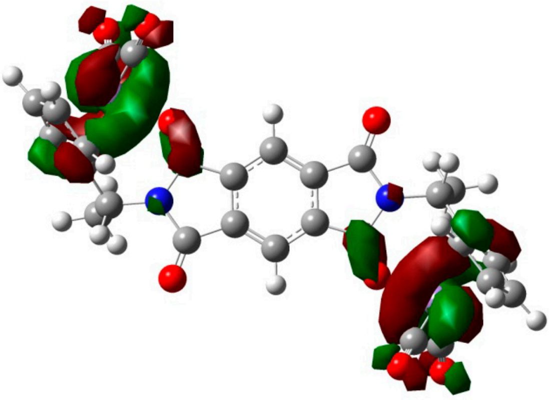 → 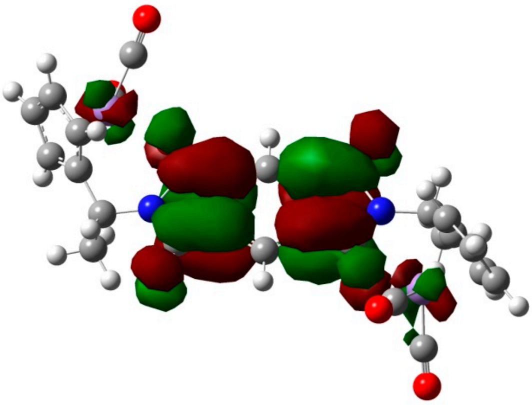
3	826	0.28189	154→157 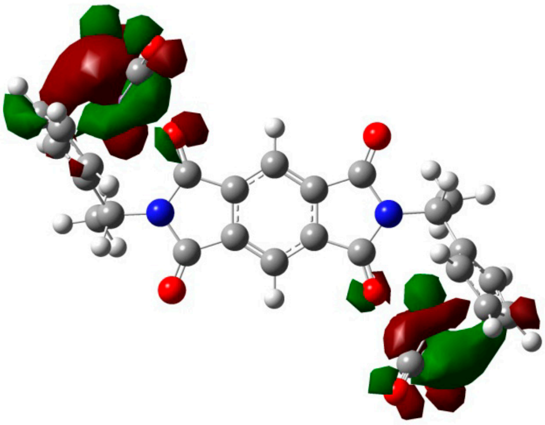 → 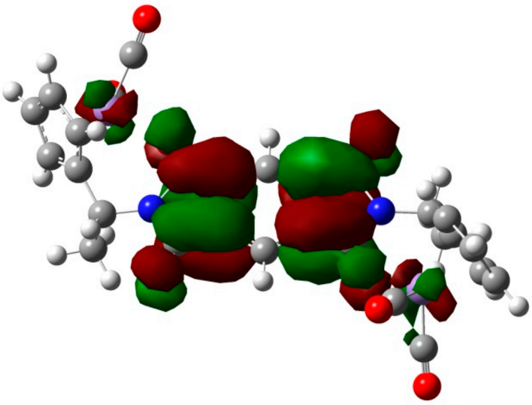
11	501	0.04114	156→159

			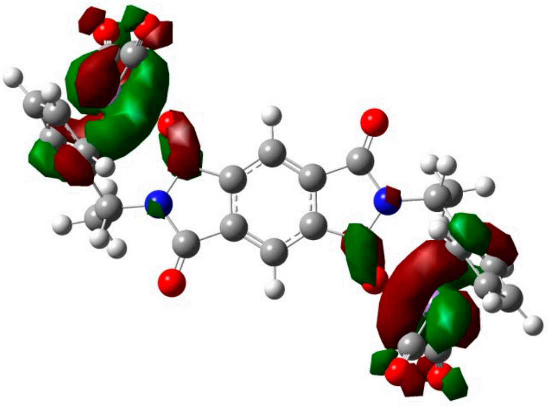 → 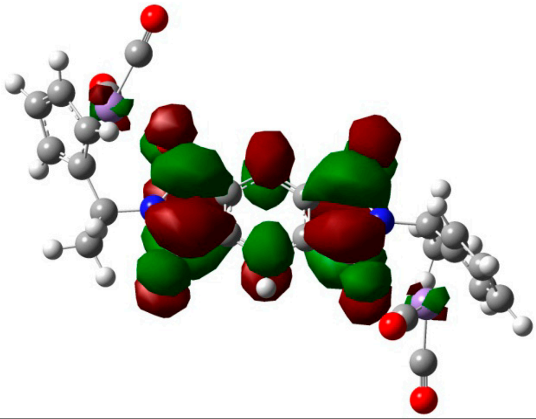
20	399	0.01508	153→157 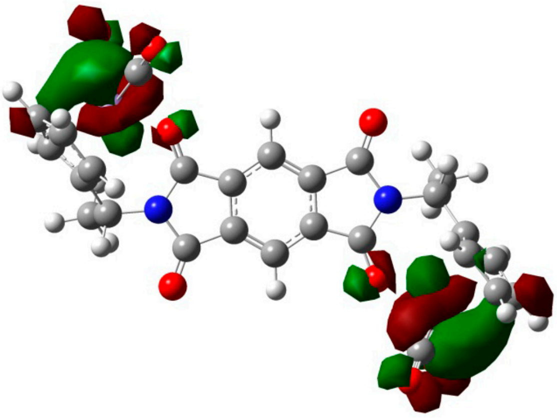 → 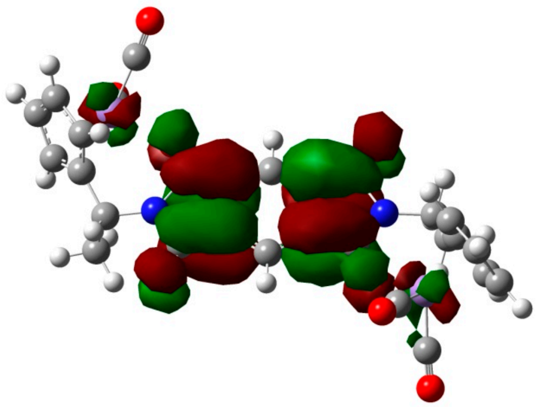
**3a**			
9	402	0.013651	173→176

			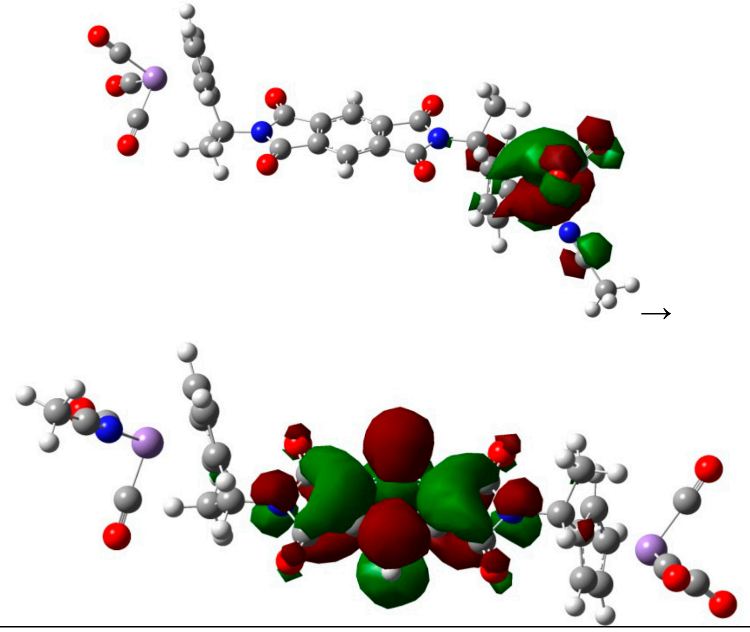

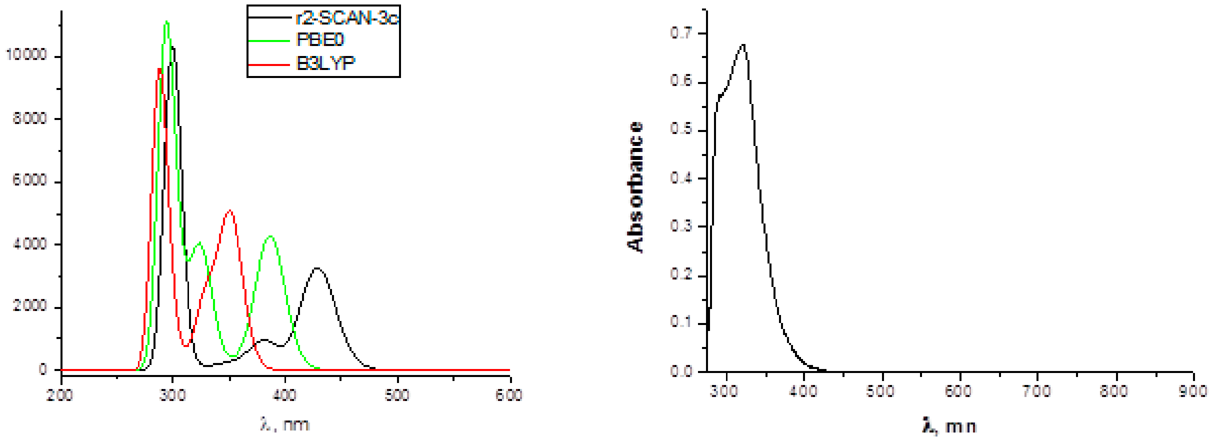

Figure S24. Comparison for TD-DFT simulated absrobtion spectra for **1** using B3LYP, r2-SCAN-3c, and PBE0 functionals (right graph); experimental absorbtion spectrum of **1** in benzene (left graph).

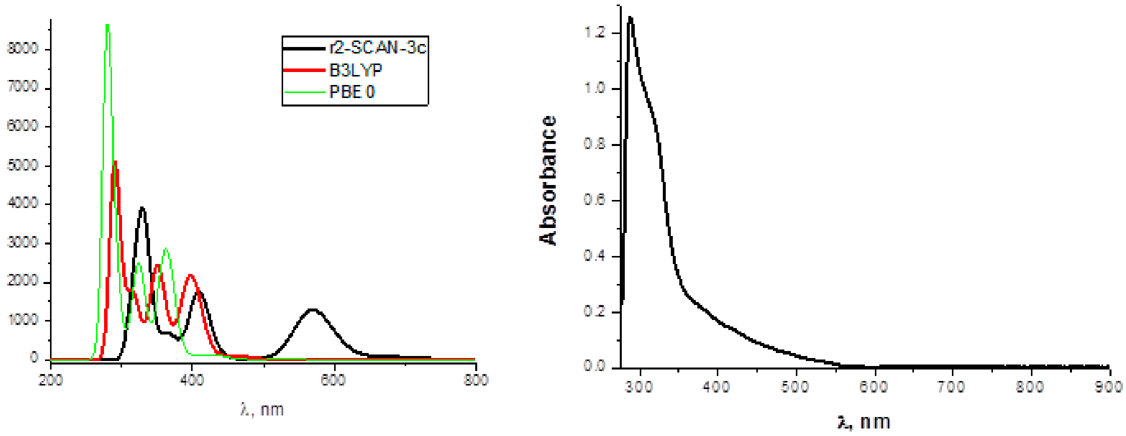

Figure S25. Comparison for TD-DFT simulated absorbtion spectra for **3a** using B3LYP, r2-SCAN-3c, and PBE0 functionals (right graph); experimental absorbtion spectrum for **3** in a mixture of benzene: acetonitrile (left graph).

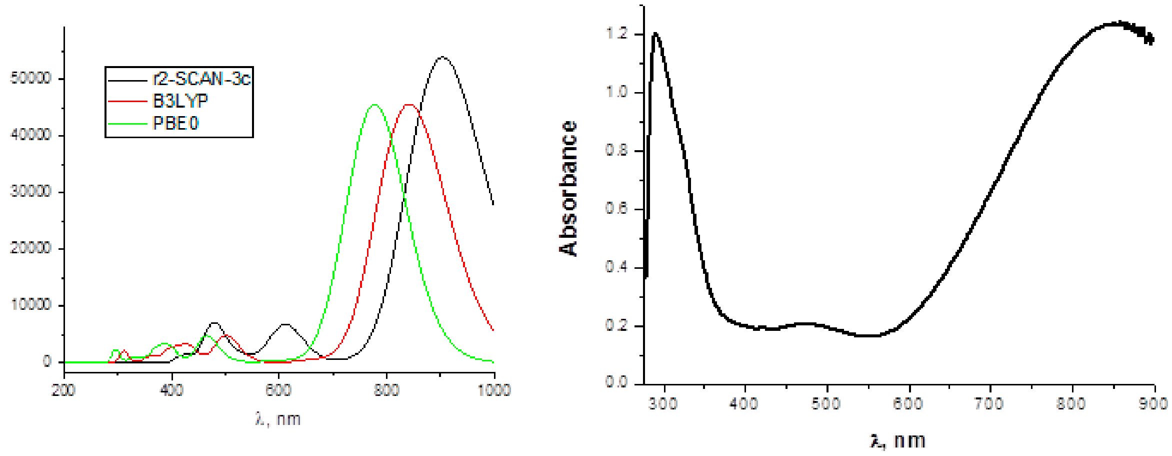

Figure S26. Comparison for TD-DFT simulated absorbtion spectra for **2a** using B3LYP, r2-SCAN-3c, and PBE0 functionals (right graph); experimental absorbtion spectrum for **2** in benzene (left graph).

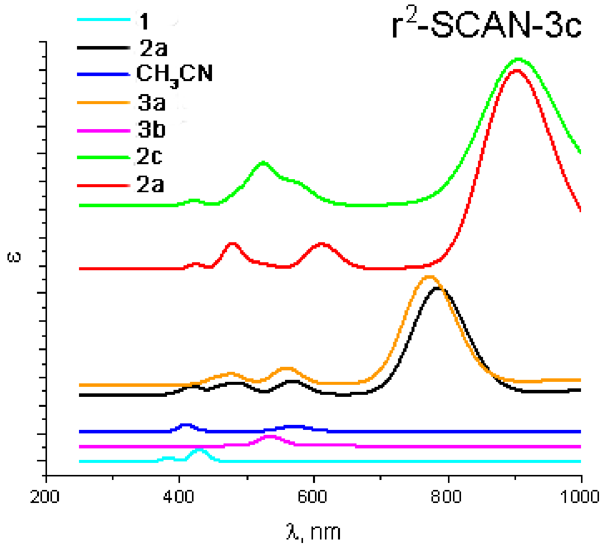

Figure S27. TD-DFT simulated absorbtion r2-SCAN-3c/Def2-TZVP level.
